# Predicted Effects of Patient Variability and Notch Signaling on In Situ Vascular Tissue Engineering

**DOI:** 10.1007/s10439-025-03843-7

**Published:** 2025-11-08

**Authors:** Jordy G. M. van Asten, Cecilia M. Sahlgren, Jay D. Humphrey, Tommaso Ristori, Sandra Loerakker

**Affiliations:** 1 Department of Biomedical Engineering, Eindhoven University of Technology, Eindhoven, the Netherlands; 2 Institute for Complex Molecular Systems, Eindhoven University of Technology, Eindhoven, the Netherlands; 3 Faculty of Science and Engineering, Biosciences, Åbo Akademi, Turku, Finland; 4 Department of Biomedical Engineering, Yale University, New Haven, CT USA

**Keywords:** Vascular tissue engineering, Computational modeling, Notch signaling, Growth and remodeling, Mechanosensing

## Abstract

In situ vascular tissue engineering aims to create living blood vessel replacements from biodegradable scaffolds. The functionality of these tissue-engineered vascular grafts (TEVGs) has often been limited, with substantial failure rate and outcome variability. Current optimization strategies seem unable to satisfy all requirements for functional TEVGs and the key sources of outcome variability remain unclear. Here, we computationally explored potential sources of TEVG variability and effects of manipulating Notch, a key vascular signaling pathway. We simulated the evolution of a TEVG from a degradable scaffold under varying patient-specific conditions, driven by immuno-mechano-mediated growth and remodeling mechanisms including Notch. Our simulations suggest that differential inflammatory production, scaffold degradation, and scaffold axial pre-stretch are major sources of variability in TEVG outcome. Immobilizing Jagged ligands to the scaffold did not substantially reduce outcome variability in our simulations, but did improve some aspects of TEVG functionality. This intervention may therefore be beneficial in combination with other treatments that compensate for predicted negative effects. Overall, our model may advance future TEVG optimization by incorporating Notch manipulations under various patient-specific conditions.

## Introduction

In situ vascular tissue engineering aims to create functional living blood vessels with an ability to grow and remodel, leveraging the patient’s body regenerative potential. These tissue-engineered vascular grafts (TEVGs) can serve as replacements or bypasses for diseased or damaged vessels in patients suffering from vascular diseases [1]. The process of in situ vascular tissue engineering involves implanting a biodegradable, synthetic scaffold directly at the functional site in the body [2–5]. This scaffold temporarily takes over vascular function, while host cells infiltrate and guide the formation of new autologous tissue as the scaffold degrades [4, 6]. TEVG luminal radius, compliance, and composition are crucial factors to estimate graft functionality [7–13]. Promising results have been obtained, as demonstrated by patent grafts that did not display major complications [14–16]. However, progression toward the clinic is still hampered by several remaining challenges, such as aneurysmal dilatation or rupture [8, 17–19]. These challenges are exacerbated by the strong variability of the results in terms of tissue functionality, the sources of which remain unclear [20–23]. Current in situ tissue engineering strategies, therefore, seem unable to satisfy all the requirements for functional TEVGs and further optimization is necessary.

Efforts to optimize TEVG functionality often focus on the structural and mechanical properties of the implanted synthetic scaffolds, such as their stiffness, anisotropy, porosity, and degradation kinetics [12, 24–26]. Given the myriad scaffold parameters and possible configurations, computational models are well suited to perform rational and systematic optimizations of scaffold design [12, 17, 27]. Despite this, it has proved challenging to identify optimal structural and mechanical scaffold properties that satisfy all aspects of TEVG functionality [12]. In addition, TEVG performance depends on the intended application. For example, while good results are commonly obtained in low pressure venous environments [22, 28], TEVGs in high-pressure arterial environments demonstrate a greater incidence of aneurysm and graft rupture [17]. This is especially the case after complete scaffold degradation when new tissue formation is insufficient. Previous simulations have predicted that altering scaffold stiffness or degradation rate alone are not effective to overcome these issues [17]. Therefore, additional optimization strategies should be developed.

The search for optimal scaffolds is further complicated by the considerable variability between patients due to age or comorbidities, including hypertension and diabetes. These factors are known to affect the remodeling capacity and inflammatory response [29] and can therefore lead to variable and suboptimal TEVG functionality. This would call for the design of patient-specific scaffolds, tailored to requirements of individual cases. The resulting additional demands placed on scaffold design would render the already difficult optimization of structural and mechanical properties of scaffolds even more challenging. This re-emphasizes the need for additional methods of optimizing TEVG performance. For example, by manipulating cellular signaling mechanisms to direct growth and remodeling (G&R) phenomena, such as collagen deposition and the proliferation of smooth muscle cells (SMCs). Such manipulations may thereby directly improve TEVG properties that are key to vascular functionality [8].

A crucial cellular signaling mechanism in vascular G&R is the Notch pathway, in which Notch receptors interact with Jagged and Delta ligands in vascular SMCs [30, 31]. Notch is regulated by mechanical stimuli, such as cyclic strain [32–34]. We have previously suggested, via simulations, that this mechano-regulation of Notch influences vascular homeostasis and G&R [33, 35, 36]. Additional simulations have predicted that vascular G&R in hypertension can be controlled by manipulating the Notch pathway, for example by altering the expression of Notch receptors [36] or by introducing external Jagged ligands [35]. Such increased control over vascular G&R could help to overcome some of the challenges of in situ vascular tissue engineering. Indeed, Notch has previously been suggested and explored as a target for tissue engineering [8, 37–39], for example, via immobilization of Jagged to biomaterials [40–42]. Therefore, we hypothesize that manipulating Notch can act as a supplementary tool to improve the functionality of TEVGs by controlling vascular G&R.

Here, we explored the potential role of Notch manipulations in improving TEVG functionality by adopting a computational approach. Previously, we simulated vascular G&R driven by a combination of mechanistic stimuli, determined by Notch signaling, and phenomenological stimuli, determined by deviations from mechanical homeostasis [35]. As the previous framework focused on native vessels rather than TEVGs, we extended our model in the present study by considering a biodegradable scaffold and a subsequent foreign body (inflammatory) response, following previous computational approaches [43, 44]. This enabled us to simulate the remodeling of a synthetic, biodegradable scaffold into a tissue-engineered vessel with G&R mediated by inflammation, mechanical homeostasis, and Notch signaling. To account for the influence of patient- and case-specific variations, we applied this model to simulate the following scenarios:

1. Variability in inflammatory production caused by differences in immune response of patients (e.g., due to aging).
2. Variability in scaffold degradation also caused by differences in immune response of patients.
3. Differential scaffold pre-stretches imposed during surgery.

To determine the effects of Notch manipulations on TEVG functionality in these scenarios, we simulated the introduction of immobilized Jagged ligands and focused on the resulting trends in luminal radius, area compliance, and tissue composition.

## Methods

### Concepts and Governing Equations

In this study, we extended a previous computational framework, describing the G&R of native blood vessels [35] by including a degradable scaffold and accounting for effects of inflammation to simulate the development of a TEVG [12, 27, 43, 44]. We also included a time delay in tissue production to account for the time necessary for cells to infiltrate the initially cell-free scaffold and initiate production.

**Fig. 1:**
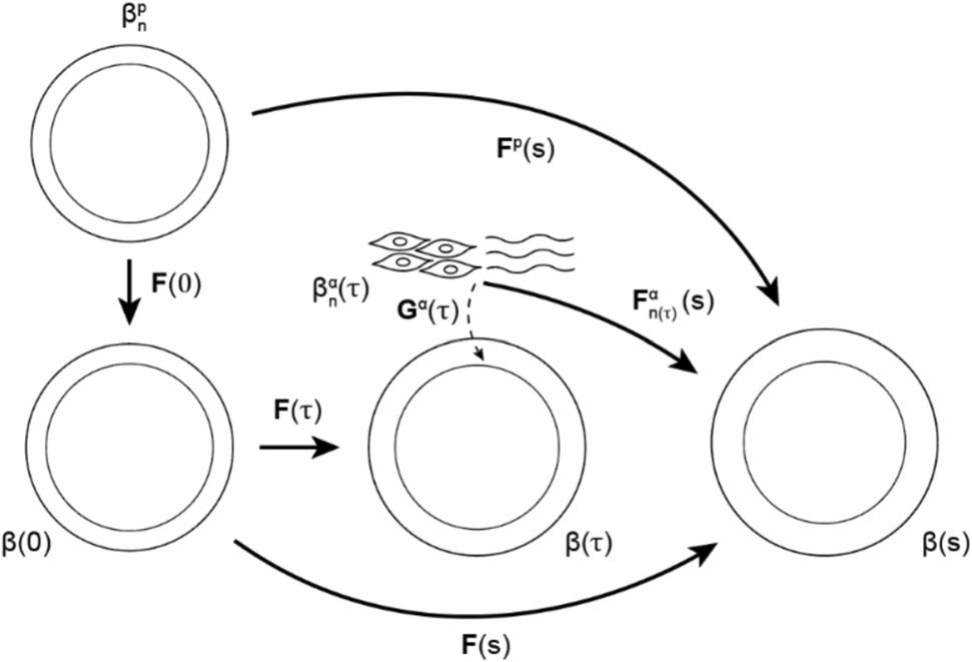
Schematic representation of the evolving geometry and kinematics of a TEVG in the computational framework. Note that polymer alone exists in the original reference configuration, with neotissue deposited thereafter relative to a series of evolving natural configurations

G&R is driven by both phenomenological factors, based on stress homeostasis and inflammation, and mechanistic factors, based on mechanosensitive Notch signaling. We adopted the constrained mixture theory [45, 46] and modeled vascular tissue as a mixture of circumferentially oriented smooth muscle and two families of collagen fibers oriented within the circumferential-axial plane. During each G&R time step $\Delta \tau$, constituent mass can be added to or removed from the mixture. The referential mass density ${\rho }_{R}^{\alpha }$ of each constituent $\alpha$ (i.e., the mass of the constituent per unit reference volume of the mixture) at the current G&R time $s$ is therefore computed as

1 $${\rho }_{R}^{\alpha }\left(s\right)={\int }_{-\infty }^{s}{m}_{R}^{\alpha }\left(\tau \right){q}^{\alpha }\left(s,\tau \right)d\tau ,$$

where ${m}_{R}^{\alpha }\left(\tau \right)$ is the referential mass production rate of constituent $\alpha$ at time $\tau$ and ${q}^{\alpha }\left(s,\tau \right)\in [\mathrm{0,1}]$ is the fraction of constituent $\alpha$ deposited at time $\tau$ that survives until time $s$. Similarly, the constituent-specific strain energy functions per unit reference volume of the mixture (${W}_{R}^{\alpha }$) are given by

2 $${W}_{R}^{\alpha }\left(s\right)=\frac{1}{ \rho }{\int }_{-\infty }^{s}{m}_{R}^{\alpha }\left(\tau \right){q}^{\alpha }\left(s,\tau \right){\widehat{W}}^{\alpha }\left({\mathbf{C}}_{n\left(\tau \right)}^{\alpha }\left(s\right)\right)d\tau ,$$

with $\rho$ the mass density of the mixture (assumed constant) and ${\widehat{W}}^{\alpha }$ the strain energy function of constituent $\alpha$ per unit volume of that constituent. The right Cauchy-Green tensor is defined as ${\mathbf{C}}_{n\left(\tau \right)}^{\alpha }\left(s\right)={\mathbf{F}}_{n\left(\tau \right)}^{\alpha T}\left(s\right){\mathbf{F}}_{n\left(\tau \right)}^{\alpha }\left(s\right)$, where ${\mathbf{F}}_{n\left(\tau \right)}^{\alpha }\left(s\right)=\mathbf{F}\left(s\right){\mathbf{F}}^{-1}\left(\tau \right){\mathbf{G}}^{\alpha }$ describes the deformation of constituent $\alpha$ from its natural (i.e., stress free) configuration at the time of deposition ${\beta }_{n}^{\alpha }(\tau )$ to the current configuration of the mixture $\beta (s)$, see Fig. 1. Here, $\mathbf{F}\left(s\right)$ is the deformation gradient tensor of the mixture with respect to the reference configuration $\beta (0)$ and the deposition stretches of the tissue constituents are accounted for via ${\mathbf{G}}^{\alpha }$. The mechanical response of smooth muscle and collagen was modeled using a Fung-type function.

The mass production of tissue constituents is driven by both Notch signaling activity and (deviations from) stress homeostasis. In particular, the production rates are given by

3 $${m}_{R}^{\alpha }\left(\tau \right)=\left(1-{e}^{-\tau }\right)\left[{m}_{nom}^{\alpha }\left(\tau \right){\Upsilon }^{\alpha }\left(\tau \right)+{m}_{h}^{\alpha }{\Upsilon }_{i}^{\alpha }(\tau )\right].$$

The term $1-{e}^{-\tau }$, represents the delay for cellular infiltration, similar to prior models [12, 27, 43, 44], and ${m}_{nom}^{\alpha }{=k}^{\alpha }\left(\tau \right){\rho }_{R}^{\alpha }\left(\tau \right)$ are nominal production rates, which depend on degradation rates ${k}^{\alpha }(\tau )$ and referential mass densities ${\rho }_{R}^{\alpha }\left(\tau \right)$. ${\Upsilon }^{\alpha }$ are constituent-specific stimulus functions defined as follows:

4 $${\Upsilon }^{\alpha }\left(\tau \right)={\Upsilon }_{N}^{\alpha }\left(\tau \right)+{\Upsilon }_{\upsigma }^{\alpha }\left(\uptau \right)+{\Upsilon }_{{\tau }_{w}}^{\alpha }\left(\tau \right)-2.$$

Here, ${\Upsilon }_{N}^{\alpha }$ captures the contribution of Notch signaling, ${\Upsilon }_{\upsigma }^{\alpha }$ that of intramural stress, ${\Upsilon }_{{\tau }_{w}}^{\alpha }$ that of wall shear stress. The latter two stimulus functions are determined by normalized deviations in intramural stress ($\Delta \sigma \left(\tau \right)$) and wall shear stress ($\Delta {\tau }_{w}\left(\tau \right)$) from homeostatic target values [47], as follows:

5 $${\Upsilon }_{\sigma }^{\alpha }\left(\tau \right)=1+{K}_{\sigma }^{\alpha }\Delta \sigma \left(\tau \right),$$

6 $${\Upsilon }_{{\tau }_{w}}^{\alpha }\left(\tau \right)=1-{K}_{{\tau }_{w}}^{\alpha }\Delta {\tau }_{w}\left(\tau \right),$$

with ${K}_{\sigma }^{\alpha }$ and ${K}_{{\tau }_{w}}^{\alpha }$ constituent-specific gain parameters. The Notch stimulus functions for smooth muscle, ${\Upsilon }_{N}^{m}$, and collagen, ${\Upsilon }_{N}^{c}$, are driven by the level of Notch signaling activity $I$ (note that these stimuli take on a value of 1 at a baseline Notch activity of 1):

7 $${\Upsilon }_{N}^{m}={B}_{1}^{m}{e}^{-{B}_{2}^{m}I},$$

8 $${\Upsilon }_{N}^{c}={B}^{c}\left(I-1\right)+1,$$

with parameters ${B}_{1}^{m}$, ${B}_{2}^{m}$, and ${B}^{c}$ based on experimentally identified correlations [35]. The level of Notch activity is determined with a computational Notch model [33, 48] which simulates interactions between Notch receptors, Jagged ligands, and Delta ligands in SMCs populating the vascular wall using a system of ordinary differential equations. The mechanosensitive regulation of Notch and Jagged production in response to SMC stretch ${\lambda }_{\theta }$ is included [33, 35], as well as the influence of immobilized Jagged ligands, ${J}_{im}$ [35, 38].

The in situ implantation of a synthetic scaffold invokes a host inflammatory response that plays an important role in the regenerative process [6]. Therefore, in the present study, we included inflammation-mediated tissue production in the model. The term ${m}_{h}^{\alpha }{\Upsilon }_{i}^{\alpha }\left(\tau \right)$ in Eq. (3) captures the inflammation-mediated production, formulated as basal mass production rates, ${m}_{h}^{\alpha }$, modulated by inflammatory stimulus functions, ${\Upsilon }_{i}^{\alpha }$, analogous to the mechano- and Notch-mediated production in the term ${m}_{nom}^{\alpha }\left(\tau \right){\Upsilon }^{\alpha }\left(\tau \right)$. The values of ${m}_{h}^{\alpha }$ are constant and represent the homeostatic mass production rate. The inflammatory stimulus functions are given as

9 $${\Upsilon }_{i}^{\alpha }={K}_{i}^{\alpha }\Delta \xi \left(\tau \right)$$

with the inflammatory burden $\Delta \xi$ prescribed phenomenologically using a gamma distribution [12, 27, 43, 44]:

10 $$\Delta \xi \left(\tau \right)=\frac{\tau }{a}\mathrm{exp}\left(1-\frac{\tau }{a}\right).$$

Parameter ${K}_{i}^{\alpha }$ is the inflammatory gain and $a$ indicates the time of peak inflammation. Note that, over time, the delay term approaches 1 and the inflammatory production vanishes such that tissue production matches the nominal production rate, once mechanical homeostasis is reached (i.e., ${\Upsilon }^{\alpha }(\tau )=1$).

### Scaffold

To capture the in situ remodeling of a TEVG from a degradable scaffold, the scaffold material, degradation kinetics, and mechanical response were here added to the original model [35]. Similar to previous studies [12, 27, 43, 44], we modeled the scaffold as a porous polymeric material, whose pores initially fill with a filler material (to enable the numerical implementation of a porous material as a continuum):

11 $${\rho }_{R}^{sc}={\rho }_{R}^{p}+{\rho }_{R}^{f}.$$

Here, $sc$ refers to the complete scaffold and $p$ and $f$ to the new constituents polymer and filler, respectively. Degradation of the polymer was captured with a sigmoidal survival function ${Q}^{p}$, as in previous models [12, 27, 43, 44]:

12 $${Q}^{p}\left(s\right)=\frac{1+\mathrm{exp}({k}^{p}{\zeta }^{p})}{1+\mathrm{exp}(-{k}^{p}\left(s-{\zeta }^{p}\right))},$$

where ${k}^{p}$ is a degradation rate parameter and ${\zeta }^{p}$ a shape parameter. We assumed that the filler is removed following the same survival function and parameters. In addition, the immobilized Jagged ligands, ${J}_{im}$, are attached to the scaffold, so their content is assumed to decrease at the same rate as scaffold degradation: ${J}_{im}\left(s\right)={J}_{im}\left(0\right){Q}^{p}(s)$.

During implantation, the scaffold may be pre-stretched in the axial direction, whether unintentionally [21] or by design. Moreover, after implantation, the scaffold is loaded by blood pressure. The associated deformations of pre-stretch and loading are captured in $\mathbf{F}(0)$ (Fig. 1). Only the polymer components are assumed to contribute mechanically at the time of implant, following a Neo-Hookean material model [12, 27, 43, 44]:

13 $${\widehat{W}}^{p}={\mu }^{p}\left(s\right)\left(\mathrm{tr}\left({\mathbf{C}}^{p}\right)-3\right),$$

where ${\mu }^{p}$ is the polymer shear modulus and ${\mathbf{C}}^{p}={\mathbf{F}}^{pT}(s){\mathbf{F}}^{p}(s)$ the right Cauchy-Green tensor derived from ${\mathbf{F}}^{p}(s)=\mathbf{F}\left(s\right)\mathbf{F}(0)$ which represents the deformation of the polymer from its natural configuration, ${\beta }_{n}^{p}(0)$, to the current configuration of the mixture, $\beta (s)$ (Fig. 1). This leads to the following Cauchy stress contribution of the polymer:

14 $${{\boldsymbol{\sigma}}}^{p}\left(s\right)=\frac{2}{\rho J\left(s\right)}{\mu }^{p}{\rho }_{R}^{p}\left(0\right){Q}^{p}\left(s\right){\mathbf{F}}^{p}\left(s\right){\mathbf{F}}^{pT}\left(s\right).$$

The shear modulus was assumed to decrease as the polymer degrades, as suggested by microstructural arguments of porous scaffolds [27, 44, 49]:

15 $${\mu }^{p}\left(s\right)=0.03{E}^{p}{\left({\Phi }^{p}\left(s\right)\right)}^{2},$$

where ${E}^{p}$ is a Young’s-type modulus for the solid polymer and ${\Phi }^{p}(s)={\rho }_{R}^{p}(s)/\rho$ the volume fraction of the polymer.

### Target Variables for Mechanical Homeostasis and Notch Signaling

As seen in Eq. (5), mechano-mediated production is partly driven by deviations in intramural stress from a homeostatic target value:

16 $$\Delta \sigma \left(\tau \right)=\frac{\widetilde{\sigma }\left(\tau \right)-{\widetilde{\sigma }}_{o}}{{\widetilde{\sigma }}_{o}},$$

with $\widetilde{\sigma }$ a scalar measure of intramural stress, representing the target variable of mechanical homeostasis, and subscript $o$ referring to the homeostatic value. In our previous model [35], this target variable was the intramural stress of the entire mixture, which then consisted of only biological material. In the current formulation, the mixture consists of both synthetic polymer and biological constituents. This requires a different definition of the target variable as polymer stress should not influence mechano-mediated production. Therefore, following the assumption that tissue production is driven by the SMCs, we used the stress experienced by the SMCs as target variable. Note that we do not know which stress levels cells actually experience but assume that they can sense their local environment via ligand-specific integrins. For this, we calculated the Cauchy stress of SMCs at the constituent level (i.e., derived from the strain energy per unit volume of the constituent):

17 $${\widehat{{\boldsymbol{\sigma}}}}^{m}\left(s,\tau \right)=\frac{1}{\mathrm{det}\left({\mathbf{F}}_{n\left(\tau \right)}^{m}\left(s\right)\right)}{\mathbf{F}}_{n\left(\tau \right)}^{m}\left(s\right){\widehat{\mathbf{S}}}^{m}\left({\mathbf{C}}_{n\left(\tau \right)}^{m}\left(s\right)\right){\mathbf{F}}_{n\left(\tau \right)}^{mT}\left(s\right),$$

with the second Piola–Kirchhoff stress at the constituent level:

18 $${\widehat{\mathbf{S}}}^{m}\left({\mathbf{C}}_{n\left(\tau \right)}^{m}\left(s\right)\right)=2\frac{\partial {\widehat{W}}^{m}\left({\mathbf{C}}_{n\left(\tau \right)}^{m}\left(s\right)\right)}{\partial \left({\mathbf{C}}_{n\left(\tau \right)}^{m}\left(s\right)\right)}.$$

Implementing the following Fung-type material model for SMCs [35]

19 $${\widehat{W}}^{m}\left({\lambda }_{n\left(\tau \right)}^{m}\left(s\right)\right)=\frac{{c}_{1}^{m}}{4{c}_{2}^{m}}\left(\mathrm{exp}\left[{c}_{2}^{m}{\left({\lambda }_{n\left(\tau \right)}^{m2}\left(s\right)-1\right)}^{2}\right]-1\right),$$

gives an expression for ${\widehat{\mathbf{S}}}^{m}$, which can be substituted into Eq. (18):

20 $${\widehat{\mathbf{S}}}^{m}\left({\mathbf{C}}_{n\left(\tau \right)}^{m}\left(s\right)\right)={c}_{1}^{m}\left({\lambda }_{n(\tau )}^{m2}-1\right)\mathrm{exp}\left[{c}_{2}^{m}{\left({\lambda }_{n\left(\tau \right)}^{m2}-1\right)}^{2}\right]{\mathbf{a}}^{m}\otimes {\mathbf{a}}^{m}.$$

Here, ${\mathbf{a}}^{m}$ is the unit vector in the direction of the SMCs, i.e., the circumferential direction, and ${c}_{1}^{m}$ and ${c}_{2}^{m}$ are material parameters of the SMCs. Therefore, the SMCs only experience intramural stress in the circumferential direction, providing us with the required scalar target variable $\widetilde{\sigma }$. This choice of target variable implicitly incorporates the phenomenon of stress-shielding [50] into the model. When the scaffold dominates the TEVG composition, the very stiff polymer bears most of the stress, thereby shielding the SMCs from stress. As the scaffold degrades, the SMCs experience higher levels of stress and are thus stimulated to contribute to mechano-mediated production.

The mechanical behavior of collagen was incorporated analogous to that of SMCs (Eqs. (17)–(20)) but substituting the stiffness parameters with collagen-specific material parameters ${c}_{1}^{c}$ and ${c}_{2}^{c}$. Note that collagen stresses were not included in the target variable for mechanical homeostasis (Eq. (16)).

Notch-mediated production is regulated by Notch activity (Eqs. (7) and (8)), which is in turn influenced by the SMC stretch in circumferential direction ${\lambda }_{\theta }$. In our previous study [35], this SMC stretch was calculated by taking the mean of the stretches of the 2000 most recently deposited cohorts of SMCs. To increase accuracy, in the present study, we adopted a weighted average that considers the relative mass density of each of the cohorts at current time $s$:

21 $${\lambda }_{\theta }\left(s\right)=\frac{{\int }_{-\infty }^{s}{\lambda }_{n(\tau )}^{m}\left(s\right){m}_{R}^{m}\left(\tau \right)q\left(s,t\right)d\tau }{{\rho }_{R}^{m}(s)}$$

where ${\lambda }_{n\left(\tau \right)}^{m}(s)$ is the SMC stretch at time $s$ of the cohort deposited at time $\tau$ with respect to its natural configuration, corresponding to the circumferential component of ${\mathbf{F}}_{n\left(\tau \right)}^{m}(s)$ (Fig. 1).

### Parameter Specification

Although the above equations are general, we focus our implementation and parameters on an interposition TEVG in a murine infrarenal abdominal aorta (IAA). The corresponding parameters of the extended model are listed in Table 1, along with their values and motivations. The gain parameters for mechano-mediated production, ${K}_{\sigma }^{\alpha }$ and ${K}_{{\tau }_{w}}^{\alpha }$, have changed compared to our previous model [35] to account for the new target variable for intramural stress (Eqs. (17)–(21)). We assumed that mechano-mediated production in TEVGs is comparable to that during the adaptation of hypertensive arteries. Therefore, the gain parameters were fitted to experimental data of hypertensive murine IAA [51], following the same procedure as before [35].

**Table 1:** Model parameter values and motivation

Symbol	Value (unit)	Description	Motivation
$[{k}^{p},{\zeta }^{p}]$	[0.12, 24] (−)	Degradation rate and shape parameters polymer	Taken from Ref. [43], based on in vivo degradation of a combined PGA & P(CL/LA) polymer scaffold in mice from Ref. [52].
${E}^{p}$	3.79 (GPa)	Young’s-type modulus polymer	Weighted average of the moduli of PGA and P(CL/LA) [12, 44, 53, 54]. Weights based on the volume ratios of these polymers in a previously used scaffold [44].
$[{\rho }_{R}^{p}\left(0\right), {\rho }_{R}^{f}(0)]$	[0.215, 0.785] ∙ $\rho$ (kg/m^3^)	Initial referential mass densities of scaffold polymer and scaffold filler	Based on experimentally determined initial porosity of a PGA-P(CL/LA) scaffold [55].
$\rho$	1050 (kg/m^2^)	Spatial mass density of the mixture	[56]
$[{m}_{h}^{m},{m}_{h}^{c}]$	[0.513, 0.468] (kg/m^2^/day)	Homeostatic mass production rates of SMCs and collagen	Found by multiplying the initial homeostatic referential mass densities of collagen and SMCs in murine IAA [35, 57] with the degradation rate parameters ${k}^{\alpha }$ of these constituents (converted to account for the difference in reference volume between a TEVG and a native vessel).
$[{k}^{m},{k}^{c}]$	[1/80, 1/80] (days)	Degradation rate parameters of SMCs and collagen.	Based on normal constituent turnover [58–60].
$a$	14 (days)	Time of peak inflammation	Based on in vivo data of macrophage infiltration in TEVGs of murine abdominal aortas [15].
$[{K}_{\sigma }^{m}, {K}_{\sigma }^{c}]$	[0.127, 2.272] (−)	Gain parameters for production of SMCs and collagen mediated by intramural stress.	Found by regression of in vivo data on hypertensive remodeling in murine IAA [51].
$[{K}_{{\tau }_{w}}^{m}, {K}_{{\tau }_{w}}^{c}]$	[0, 6.026] (−)	Gain parameters for production of SMCs and collagen mediated by wall shear stress.	Found by regression of in vivo data on hypertensive remodeling in murine IAA [51].
${K}_{i}^{m}= {K}_{i}^{c}$	5.45 (−)	Inflammatory gain parameters of SMCs and collagen.	Taken from Ref. [44] based on inflammatory gain of TEVGs in the IVC position in SCID/bg mice.
$[{B}_{1}^{m}, {B}_{2}^{m},{B}^{c}]$	[47.21, − 3.855, 0.3161] (−)	Parameters characterizing the relationship between Notch activity and the Notch stimulus functions for collagen and SMCs.	Found by regression of *in vitro* data of Notch3 receptor, collagen, and KI67 gene expressions in human coronary artery SMCs [32, 35].
$[{c}_{1}^{m}, {c}_{2}^{m}$, ${c}_{1}^{c}, {c}_{2}^{c}]$	[343 kPa, 1.23 450 kPa, 3.51]	Material parameters of SMCs and collagen.	Taken from Ref. [61], based on nonlinear regression of in vivo data from murine IAA [51].

### Simulations

To simulate scenarios with different inflammatory production, scaffold degradation, and scaffold pre-stretch, we introduced parameters ${f}_{i}$, ${f}_{d}$, and ${\lambda }_{pre}$. In particular, to vary inflammatory production, gain parameter ${K}_{i}$ was multiplied with factor ${f}_{i}$ to capture conditions of decreased (${f}_{i}<1$) and increased (${f}_{i}>1$) inflammatory production. The shape and timing of the gamma distribution for inflammatory production (Eq. (16)) were not changed. Differences in scaffold degradation were included by multiplying the degradation rate parameter ${k}^{p}$ with factor ${f}_{d}$ and dividing the degradation shape parameter ${\zeta }^{p}$ by ${f}_{d}$ to either accelerate (${f}_{d}>1$) or delay (${f}_{d}<1$) scaffold degradation. We included axial scaffold pre-stretches (${\lambda }_{pre}$) in the deformation gradient tensor $\mathbf{F}(0)$ which describes the deformation of the scaffold from its stress-free configuration ${\beta }_{n\left(\tau \right)}^{p}$ to the loaded in vivo configuration $\beta (0)$ (Fig. 1). All simulations were performed with an imposed blood pressure of 14.4 kPa.

Follow-up times of in situ vascular tissue engineering studies in mice vary considerably, from a few weeks [62] to up to 2 years [22]. Here, we used a simulation time of about six months (180 days), a common time point [43, 44, 52], ensuring that we capture G&R of the TEVG well beyond complete scaffold degradation. We evaluated TEVG functionality based on the key properties identified in the Introduction: luminal radius, compliance, and composition. The compliance $C$ was defined as the change in cross-sectional area of the TEVG ($\Delta A$) divided by the change in intramural pressure ($\Delta P$) causing this change in area: $C=\frac{\Delta A}{\Delta P}$. We introduced the metrics Mean Absolute Error (MAE) and Mean Absolute Variation (MAV) to assess effects of different scenarios on TEVG properties. The MAE of a property is defined as the absolute difference between the predicted and native values, averaged over time, and normalized to the native values. This metric was used to give an indication of the overall deviation of TEVG properties from their native values. The MAV is defined as the absolute difference between the predicted values for the extreme cases in each scenario (i.e., extreme values of ${f}_{i}$, ${f}_{d}$, and ${\lambda }_{pre}$), averaged over time, and normalized to the native values. This metric was used to give an indication of the overall variation of these properties caused by patient- and case-specific variations. The present model was solved at every G&R step, analogous to the original model [35], to obtain the evolved TEVG volume, geometry, and composition satisfying the linear momentum balance in absence of body forces.

## Results

### Predicted Evolution of TEVG Properties Is Consistent with Previous In Vivo Results

**Fig. 2:**
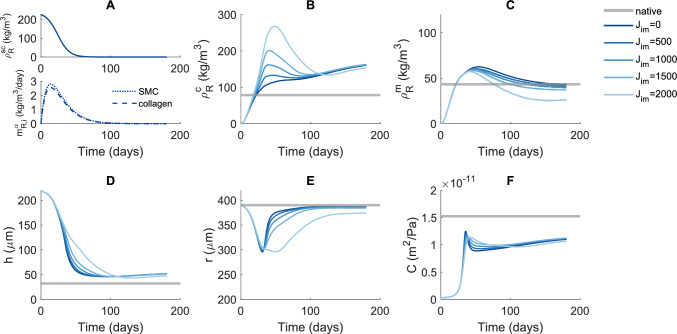
Predicted time courses of scaffold density (**A**, top), inflammatory production of collagen and SMCs (**A**, bottom), collagen referential mass density (**B**), SMC referential mass density (**C**), vessel wall thickness (**D**), luminal radius (**E**), and area compliance (**F**) of a TEVG with varying levels of immobilized Jagged (${\mathrm{J}}_{\mathrm{im}}$) compared to native values

We first simulated the evolution of a TEVG from a synthetic scaffold under reference conditions (dark blue lines in Fig. 2), corresponding to the parameter values in Table 1.

The extent to which the predicted values of TEVG properties approximate native values [57, 61, 63] varies (dark blue versus gray lines in Fig. 2). While the luminal radius maintains values close to native (Fig. 2E), both thickness and compliance approach native values but do not reach them within the simulated time frame (Fig. 2D and F). This indicates that the TEVG maintains good patency but is thicker and stiffer than a native vessel. The decrease in luminal radius is ultimately caused by scaffold degradation. In fact, this process leads to an isotropic decrease in material and thus of total volume $\mathrm{V}$, related to thickness ($\mathrm{h}$) and radius ($\mathrm{r}$) according to $\mathrm{V}=\uppi(\mathrm{hr}+\mathrm{h}^2).$ Despite an increasing compliance (Fig. 2F) would have led to an increased radius, in our simulations this isotropic decrease in volume had a dominant role, eventually leading to a decreased radius that could satisfy mechanical equilibrium. TEVG composition varies with time, with collagen becoming more dominant over time (Fig. 2B and C). This variation of composition over time is caused by a switch from an initial phase, dominated by inflammatory production, to a subsequent phase dominated by mechano-mediated production as the scaffold degrades. The inflammatory phase has similar effects on collagen and SMC content, causing both to increase. The mechano-mediated phase is characterized by increases in stress and strain, affecting the Notch and intramural stress stimuli, which have more variable effects on collagen and SMC production (Eqs. (5), (7) and (8)), resulting in a continued increase of collagen content and a switch to a decrease in SMC content.

The trends in these predictions are in line with previous in vivo and *in silico* findings in mice or rat TEVGs. In particular, a decrease in TEVG thickness after implantation is common, both in vivo and *in silico* [22, 44, 52], with values nevertheless higher than native for at least 6 months [22, 44, 52, 63, 64]. Likewise, a high collagen content has also been observed *in vivo*, with values higher than native at four weeks [62] and continuing to increase up to 6 months [18], similar to our predictions. Finally, TEVGs are commonly patent [15, 22, 63] and less compliant than native vessels in the first six months after implantation [43, 52, 63–65].

### Immobilized Jagged Affects Collagen and SMC Content in Opposing Ways

Next, we simulated TEVG evolution as influenced by different levels of Jagged ligands (${\mathrm{J}}_{\mathrm{im}}$) immobilized to the scaffold, to investigate the possible impact of this Notch manipulation. Our model predicts that effects of immobilized Jagged are dose-dependent and nonlinear with respect to the value of ${\mathrm{J}}_{\mathrm{im}}$ (Fig. 2). While luminal radius and compliance are relatively unaffected (Fig. 2E and F), immobilized Jagged has opposite effects on collagen and SMC content, which increase and decrease, respectively (Fig. 2B and C). This is expected because immobilized Jagged enhances Notch activation, leading to an increase in collagen production (Eq. (8)) and a decrease in SMC production (Eq. (7)). Another difference is found in the temporal evolution of collagen and SMCs, with transient effects of immobilized Jagged on collagen and sustained effects on SMC content (Fig. 2B and C). These effects are caused by the temporary presence of immobilized Jagged ligands, whose content decreases as the scaffold degrades. Initially, the effects of immobilized Jagged are small because both collagen and SMC production are dominated by inflammation (Fig. 2A). Then, as mechano-mediated production becomes more important, immobilized Jagged has visible effects on composition. However, as the scaffold degrades, the decreasing number of remaining immobilized Jagged ligands still has a considerable effect on SMC production, but no longer on collagen. These effects are caused by the strong exponential relationship between Notch activity and SMC proliferation (Eq. (7)) and the weaker linear relationship between Notch activity and collagen production (Eq. (8)). The transient increase in thickness induced by immobilized Jagged is consistent with the transient increase in collagen content which contributes most to TEVG volume. Together, these results reveal that immobilized Jagged may have the potential to substantially affect TEVG composition, with collagen and SMC content affected in opposing ways.

### Changes in Inflammatory Production Mainly Affect TEVG Composition and the Impact of Adding Immobilized Jagged Is Small

**Fig. 3:**
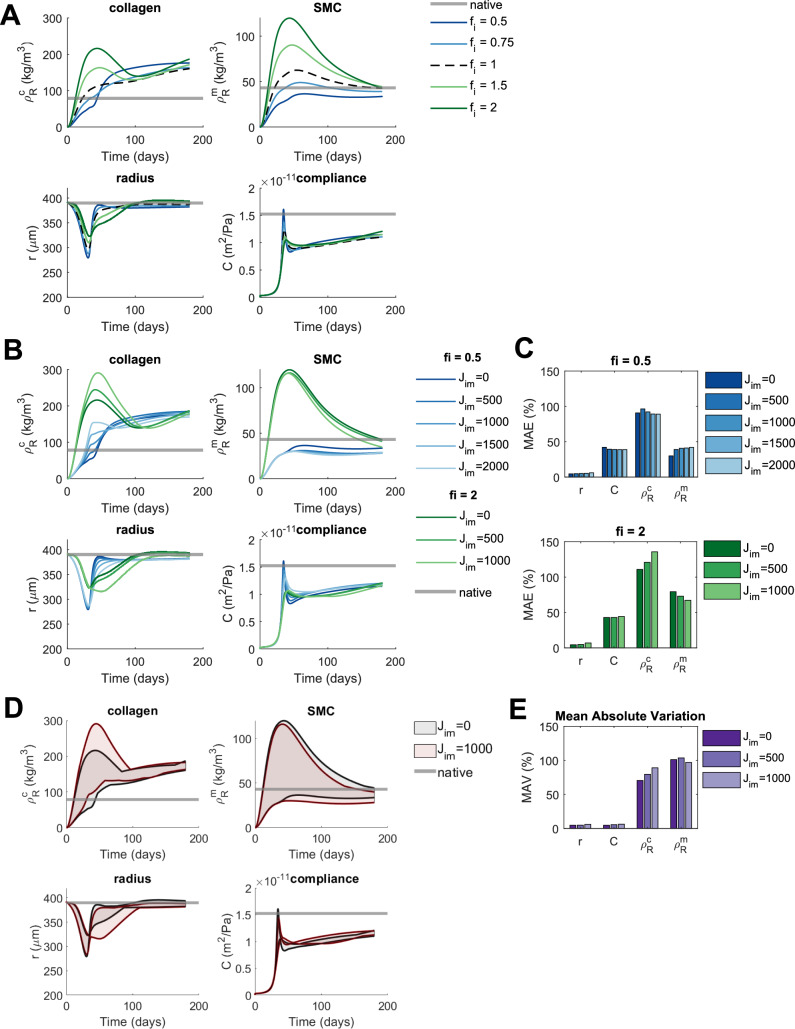
Predicted effects of changes in inflammatory production on TEVG properties including the influence of immobilized Jagged ligands (${\mathrm{J}}_{\mathrm{im}}$). **A** The evolution of TEVG properties is shown under reference conditions (${\mathrm{f}}_{\mathrm{i}} = 1$), increased inflammatory production (${\mathrm{f}}_{\mathrm{i}}> 1$) and decreased inflammatory production (${\mathrm{f}}_{\mathrm{i}}<1$) and compared to native values. **B** The effects of different levels of immobilized Jagged ligands on the evolution of TEVG properties are shown for an increase (${\mathrm{f}}_{\mathrm{i}}=2$) and a decrease (${\mathrm{f}}_{\mathrm{i}}=0.5$) in inflammatory production and compared to native values. **C** The MAE of TEVG properties with respect to their native values for an increase (${\mathrm{f}}_{\mathrm{i}}=2$) and a decrease (${\mathrm{f}}_{\mathrm{i}}=0.5$) in inflammatory production, defined as the absolute difference between the predicted and native values, averaged over time, and normalized to the native values. This gives an indication of the overall deviation of TEVG properties from their native values. **D** The variation in TEVG properties caused by changes in inflammatory production is visualized in the absence (${\mathrm{J}}_{\mathrm{im}}=0$) and presence (${\mathrm{J}}_{\mathrm{im}}=1000$) of immobilized Jagged and compared to native. For this, the minimum and maximum values of the TEVG properties at each time point for the different conditions of inflammatory production (${\mathrm{f}}_{\mathrm{i}}=[0.5, 0.75, 1, 1.5, 2]$) are plotted and the area between them is shaded. This way, the shaded areas indicate all the possible values that the TEVG properties can take on under the given changes in inflammatory production. **E** The MAV of TEVG properties for different levels of immobilized Jagged, defined as the absolute difference between predicted values for ${\mathrm{f}}_{\mathrm{i}}=0.5$ and ${\mathrm{f}}_{\mathrm{i}}=2$, averaged over time, and normalized to the native values. This gives an indication of the overall variation of these properties caused by changes in inflammatory production

To investigate effects of patient characteristics, we first looked at variations in inflammatory response, corresponding to patients with different immune systems. In our simulations, changes in inflammatory production affect mainly collagen and SMC content, while luminal radius and compliance are relatively unaltered (Fig. 3A). The effects are most prominent in early stages, consistent with the assumption that the inflammatory response peaks just after implantation (Eqs. (15) and (16)). Interestingly, while a decrease in inflammatory production initially leads to a lower collagen content, the ultimate collagen content is predicted to be higher compared to the default inflammatory production. This is caused by a higher compliance due to the reduced initial collagen production, resulting in higher cell stresses and, consequently, more stress-mediated collagen production in later stages (Eqs (4), (5) and (14)). An increase in inflammation has opposite effects: initially a higher collagen content, followed by a decrease toward the reference value.

This variability in composition caused by differences in inflammatory production cannot be compensated by immobilized Jagged. Immobilized Jagged affects TEVG functionality more under increased than decreased inflammatory production, with opposing effects on collagen content (increase) and SMC content (small decrease) (Figs 3B). These effects make collagen content less native-like when the inflammatory production is increased but not when it is decreased (Fig. 3B and C). SMC content behaves in an opposite way; becoming more native-like with immobilized Jagged for ${f}_{i}=2$ and less native-like for ${f}_{i}=0.5$. This might be due to the TEVG being less compliant under increased inflammatory production (Fig. 3A), resulting in lower stress-mediated production (Eqs. (4), (5) and (14)) and, thereby, a higher relative influence of immobilized Jagged.

Consistent with the increase in collagen content under increased inflammatory production, the variation in collagen content increases slightly under immobilized Jagged, while the variation in other properties is approximately unchanged (Fig. 3D and E). Overall, these simulations show that the effects of immobilized Jagged on TEVGs with different inflammatory production are small and do not substantially improve TEVG functionality or reduce outcome variability.

### Changes in Scaffold Degradation Have Considerable Long-Term Effects on TEVGs But These Effects Are Not Sensitive to Immobilized Jagged

Differences in patient immune response may affect not only inflammatory production but also scaffold degradation [4]. Therefore, as a next step, we investigated effects of changes in scaffold degradation profile on TEVGs, together with the impact of immobilized Jagged ligands.

**Fig. 4:**
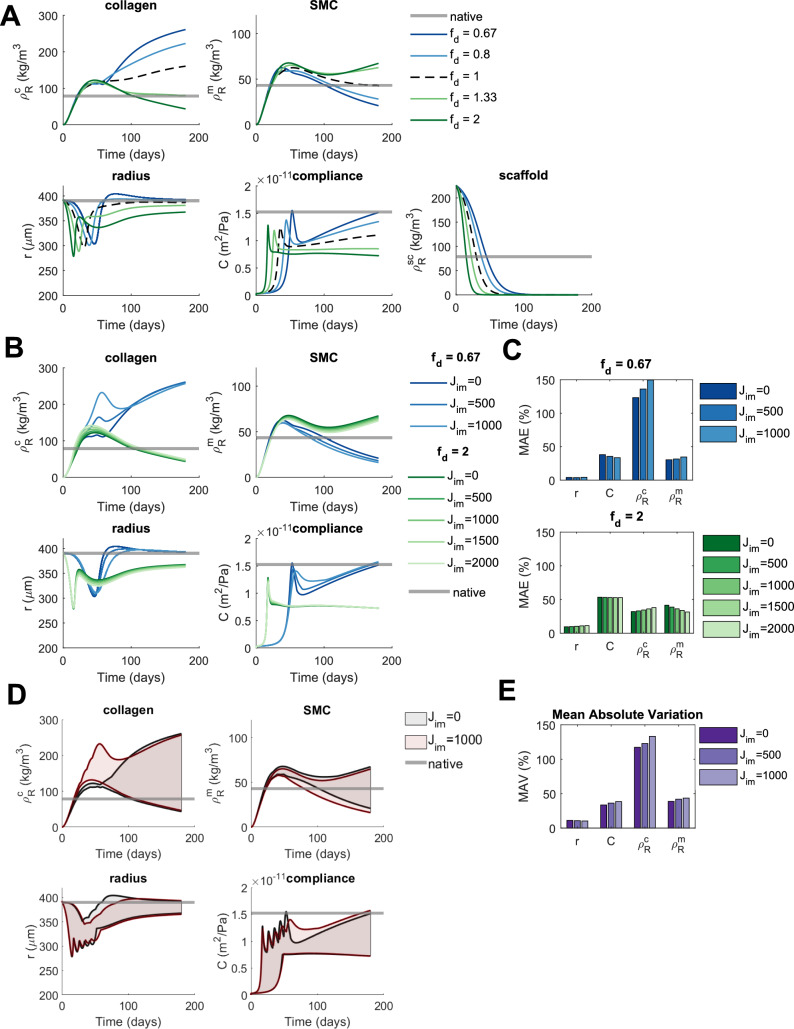
The predicted effects of changes in scaffold degradation profile on TEVG properties including the influence of immobilized Jagged ligands (${\mathrm{J}}_{\mathrm{im}}$). **A** The evolution of TEVG properties is shown under reference conditions (${\mathrm{f}}_{\mathrm{d}} = 1$), increased scaffold degradation (${\mathrm{f}}_{\mathrm{d}}> 1$), and decreased scaffold degradation (${\mathrm{f}}_{\mathrm{d}}<1$) and compared to native values. **B** The effects of different levels of immobilized Jagged ligands on the evolution of TEVG properties are shown for an increase (${\mathrm{f}}_{\mathrm{d}}=2$) and a decrease (${\mathrm{f}}_{\mathrm{d}}=0.67$) in scaffold degradation and compared to native values. **C** The MAE of TEVG properties with respect to their native values for an increase (${\mathrm{f}}_{\mathrm{d}}=2$) and a decrease (${\mathrm{f}}_{\mathrm{d}}=0.67$) in scaffold degradation, defined as the absolute difference between the predicted and native values, averaged over time, and normalized to the native values. This gives an indication of the overall deviation of TEVG properties from their native values. **D** The variation in TEVG properties caused by changes in scaffold degradation is visualized in the absence (${\mathrm{J}}_{\mathrm{im}}=0$) and presence (${\mathrm{J}}_{\mathrm{im}}=1000$) of immobilized Jagged and compared to native. For this, the minimum and maximum values of the TEVG properties at each time point for the different conditions of scaffold degradation (${\mathrm{f}}_{\mathrm{d}}=[0.67, 0.8, 1, 1.33, 2]$) are plotted and the area between them is shaded. This way, the shaded areas indicate all the possible values that the TEVG properties can take on under the given changes in scaffold degradation. **E** The MAV of TEVG properties for different levels of immobilized Jagged, defined as the absolute difference between predicted values for ${\mathrm{f}}_{\mathrm{d}}=0.67$ and ${\mathrm{f}}_{\mathrm{d}}=2$, averaged over time, and normalized to the native values. This gives an indication of the overall variation of these properties caused by changes in scaffold degradation

Changes in scaffold degradation profile have considerable long-term effects on all of the TEVG properties (Fig. 4A). These effects increase over time and persist beyond the lifetime of the scaffold. The effects on collagen and SMC content are opposite. For example, a faster scaffold degradation results in a higher initial volume loss and a corresponding decrease in luminal radius (Fig. 4A) and thickness. This thickness reduction is apparently more dominant in combination with the turnover of tissue constituents and the concomitant change in reference state. Collectively, these result in higher circumferential strains and stresses, which affect the Notch, stress, and wall shear stress stimuli, in turn leading to more SMC production and less collagen production (Eqs. (5)-(8)). Surprisingly, graft compliance increases with slower scaffold degradation, despite the presence of more collagen (Fig 4A), which is expected to lower compliance due to smaller strains. This may be attributable to the increase in luminal radius in the later stages of the simulation having a bigger effect on compliance than the stiff collagen. The opposite effect is seen with faster degradation: a decrease in compliance possibly caused by a decreased luminal radius.

Immobilized Jagged is predicted to counteract only minimally the strong effects that variations in scaffold degradation have on the long-term properties of TEVGs (Fig. 4B). The short-term effects of immobilized Jagged are slightly greater, with transient increases in collagen content and compliance predicted in slow-degrading scaffolds. Despite these short-term effects, immobilized Jagged has little influence on the MAE of TEVG properties (Fig. 4C). Only SMC content in fast-degrading scaffolds and compliance in slow-degrading scaffolds become slightly more native-like on average (Fig. 4C), while collagen content becomes slightly less native-like under all conditions. Similarly, while immobilized Jagged makes the variation (MAV) larger for all properties except luminal radius, the increase is very small (Fig. 4D and E). Overall, these simulations suggest that the impact of changes in scaffold degradation profile on TEVG properties is substantial, particularly in the long term, but affected little by the presence of immobilized Jagged. In addition, these results, together with those in “Changes in Inflammatory Production Mainly Affect TEVG Composition and the Impact of Adding Immobilized Jagged Is Small” Sect., suggest that inter-patient differences in immune response might at least partly contribute to variability in TEVG composition.

### Scaffold Pre-stretches Affect TEVG Evolution and Adding Immobilized Jagged Results in Inconsistent Effects on TEVG Properties

In addition to patient-specific characteristics like the immune response, variations in TEVG outcome may also arise from the scaffold being (inadvertently) elongated or compressed axially during implantation [21, 22], potentially changing its mechanical response and thereby mechano-regulated G&R.

**Fig. 5:**
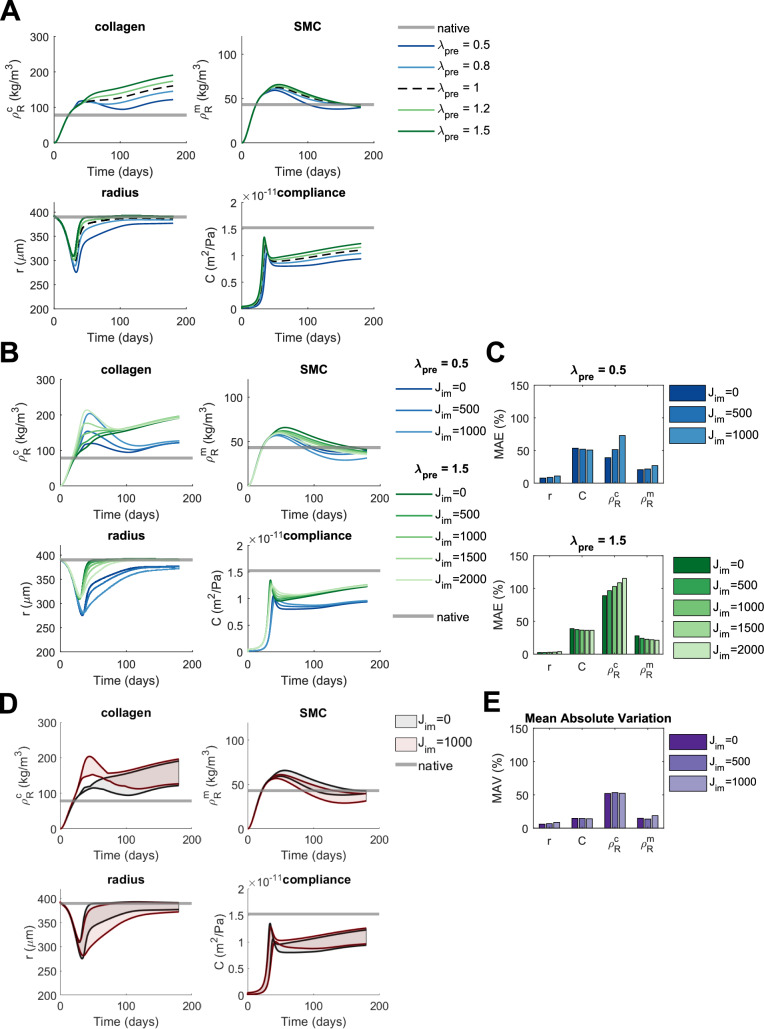
The predicted effects of changes in scaffold pre-stretch profile on TEVG properties including the influence of immobilized Jagged ligands (${\mathrm{J}}_{\mathrm{im}}$). **A** The evolution of TEVG properties is shown under reference conditions (${\uplambda }_{\mathrm{pre}} = 1$), increased scaffold pre-stretch (${\uplambda }_{\mathrm{pre}}> 1$), and decreased scaffold pre-stretch (${\uplambda }_{\mathrm{pre}}<1$) and compared to native values. **B** The effects of different levels of immobilized Jagged ligands on the evolution of TEVG properties are shown for scaffold elongation (${\uplambda }_{\mathrm{pre}}=1.5$) and compression (${\uplambda }_{\mathrm{pre}}=0.5$) and compared to native values. **C** The MAE of TEVG properties with respect to their native values for scaffold elongation (${\uplambda }_{\mathrm{pre}}=1.5$) and compression (${\uplambda }_{\mathrm{pre}}=0.5$), defined as the absolute difference between the predicted and native values, averaged over time, and normalized to the native values. This gives an indication of the overall deviation of TEVG properties from their native values. **D** The variation in TEVG properties caused by changes in scaffold pre-stretch is visualized in the absence (${\mathrm{J}}_{\mathrm{im}}=0$) and presence (${\mathrm{J}}_{\mathrm{im}}=1000$) of immobilized Jagged and compared to native. For this, the minimum and maximum values of the TEVG properties at each time point for the different conditions of scaffold pre-stretch (${\uplambda }_{\mathrm{pre}}=[0.5, 0.8, 1, 1.2, 1.5]$) are plotted and the area between them is shaded. This way, the shaded areas indicate all the possible values that the TEVG properties can take on under the given changes in scaffold pre-stretch. **E** The MAV of TEVG properties for different levels of immobilized Jagged, defined as the absolute difference between predicted values for ${\uplambda }_{\mathrm{pre}}=0.5$ and ${\uplambda }_{\mathrm{pre}}=1.5$, averaged over time, and normalized to the native values. This gives an indication of the overall variation of these properties caused by changes in scaffold pre-stretch

The simulations predicted that compressive pre-stretches (${\lambda }_{pre}<1$) have opposite effects on TEVGs compared to tensile pre-stretches (${\lambda }_{pre}>1$) (Fig. 5). In particular, compression results in decreases in collagen content, SMC content, luminal radius, and compliance, while tension causes increases in these quantities, all of which are sustained up to six months (Fig. 5A). The magnitude of the effects is not similar for all properties, however, with the most pronounced changes predicted for collagen content. The impact on collagen and SMC content is probably caused by changes in axial pre-stretch also resulting in changes in circumferential pre-stretch due to incompressibility. This biaxial coupling, in turn, affects the stiffness in the circumferential direction, putting more or less load onto the SMCs (Eq. (18)), thereby altering stress-mediated production (Eqs. (4), (5) and (14)). The changes in luminal radius are probably caused by changes in composition, with decreases in collagen and SMC for lower pre-stretches, for example, constituting a decrease in volume and the vessel finding a mechanical equilibrium at a smaller luminal radius.

Comparing predicted TEVG properties to native values after applying immobilized Jagged reveals varying trends. While collagen content and luminal radius become less native-like (Fig. 5C), mainly due to early transient changes (Fig. 5B), compliance becomes more native-like, albeit modestly (Fig. 5C) and temporarily (Fig. 5B). The effects of immobilized Jagged on SMC content depend on the type of pre-stretch: SMC content becomes more native-like for both compression and tension in early stages, but the deviation from native becomes larger over time for scaffolds with ${\lambda }_{pre}<1$ (Fig. 5B). As a result, the MAE for SMC content only decreases for ${\lambda }_{pre}>1$ (Fig. 5C). Nevertheless, the effects of immobilized Jagged are small compared to the effects of pre-stretch variations, similar to the cases of differences in inflammatory production (Fig. 3B and C) and scaffold degradation (Fig. 4B and C).

The effects of immobilized Jagged on outcome variation are small: the MAV is not affected appreciably (Fig. 5E) and there are only subtle changes in variation over time (Fig. 5D). The most notable change is an early increase in collagen variation (Fig. 5D), consistent with the transient increase in collagen production (Fig. 5B). In conclusion, pre-stretches imposed on scaffolds during implantation can affect the evolution of TEVG properties and the effects of adding immobilized Jagged are small.

## Discussion

In situ vascular tissue engineering still faces several challenges, including outcome variability [20–23] and graft failure [8, 17–19]. To overcome these challenges, optimizing only the structural and mechanical properties of scaffolds may not be sufficient, especially given patient- and case-specific variations. Therefore, here we explored sources of variability and the potential impact of interventions to the key signaling pathway Notch. To this end, we extended previous computational frameworks [35, 43, 44] to simulate the evolution of a TEVG and the role of Notch signaling in this process. Our model captured key features of TEVG evolution in agreement with previous in vivo observations [15, 18, 22, 52, 62–64, 66]. Moreover, our simulations suggested that the variability in TEVG outcome might be at least partly explained by patient- or case-specific variations, particularly changes in scaffold degradation, that have a substantial impact on transient and long-term TEVG properties. Manipulating the Notch pathway by immobilizing Jagged ligands to the scaffold was predicted to affect TEVG functionality, but without substantial improvements or reductions in outcome variability caused by patient- and case-specific conditions. Nevertheless, the current model extends our ability to understand and optimize tissue engineering strategies by considering Notch manipulations.

Our simulations suggest that patient characteristics have a great impact on TEVG outcome and illustrate how computational models can increase our understanding of the underlying mechanisms. The impact of patient-specific conditions on the functionality of TEVGs has been recognized before [4, 10, 29], but the underlying causes remain unknown. Elucidating these is clinically relevant, given the considerable variation among patients requiring vascular replacements. Here, we varied the inflammatory tissue production and scaffold degradation as examples of parameters that may be influenced by patient conditions such as age, hypertension, or diabetes. These conditions are known to affect the inflammatory response and thus, indirectly, also inflammatory-mediated scaffold degradation. Diabetic conditions have been shown to cause a switch in SMCs toward a synthetic phenotype [67–70] associated with increased SMC proliferation [68–70] and collagen production [70]. As diabetes generally leads to an increased inflammatory response, these findings are in line with our predictions that an increase in inflammatory production causes higher SMC and collagen content in the TEVGs.

A previous study has found a higher SMC content in TEVGs with fast-degrading scaffolds in vivo [24]. Our simulations agree with these findings and suggest that they may be explained by faster scaffold degradation resulting in higher stresses and strains, and thus more stress- and Notch-mediated SMC production. Overall, our results indicate that the mechano-mediated mechanisms in our computational model can capture consequences of patient-specific conditions for TEVG properties. They also suggest that changes in scaffold degradation have sustained effects on TEVG properties, while changes in inflammatory production have transient effects.

Nevertheless, our model was not able to capture the increased dilatation of arterial TEVGs experimentally observed in murine studies [17]. A possible explanation for this is that our model did not incorporate differences between the mechanical properties of newly deposited and mature ECM. To reach stiffness values representative of native arteries, deposited ECM needs to undergo a process of maturation that is currently absent in our simulations. Newly deposited collagen immediately was assumed to have the same stiffness as mature collagen, likely determining the absence of dilation in our predictions. Thus, future studies may focus on overcoming this limitation by explicitly modeling the temporal dynamics of collagen maturation.

In addition to these patient conditions, other patient-specific phenomena may also result in variations in TEVG functionality. It was previously hypothesized that scaffold axial pre-stretches are a possible source of variability in long-term TEVG functionality [21, 22]. Our results align with this hypothesis, predicting that changes in scaffold pre-stretch affect long-term TEVG properties, especially collagen content and compliance. Mechanistically, this pre-stretch also changes the circumferential stiffness of TEVGs, thereby altering stress-mediated production. Our predictions of a more native-like compliance and luminal radius with tension-related pre-stretches (${\lambda }_{pre}>1$) are in line with prior findings [21] in which better performance of pre-stretched TEVGs was observed. However, our simulations suggested caution using this strategy, as they also predicted that tensile pre-stretches made collagen content less similar to native values, potentially resulting in suboptimal performance. Therefore, future studies should investigate the potential improvements to TEVG functionality caused by positive scaffold pre-stretches. Overall, our findings indicate that scaffold pre-stretches may affect long-term TEVG outcome by changing scaffold mechanical properties and thereby altering stress-mediated growth, with positive pre-stretches predicted to make compliance and luminal radius more native-like.

Biological interventions may be adopted to counteract the predicted effects of patient-specific characteristics. Here, we investigated the potential of Jagged ligands immobilized to scaffolds. We found that the sensitivity of TEVG properties to immobilized Jagged depends on patient-specific conditions. In general, across all scenarios, our simulations predicted that immobilized Jagged only slightly affects radius and compliance, while it causes an increase in collagen and decrease in SMC content. The level of increased collagen and decreased SMC content depends highly on patient characteristics, with a reduced impact of immobilized Jagged when inflammatory production or scaffold degradation are varied relative to the reference situation. This is consistent with the finding that the efficacy of scaffold functionalization in TEVGs is reduced by effects of diabetes [71]. These results imply that biological interventions in tissue engineering should be tested under different patient conditions, as these conditions may affect not only TEVG properties, but also the efficacy of the interventions used to optimize these properties, such as Jagged immobilization to scaffolds.

Despite their dependence on patient conditions, Notch manipulations may be a useful tool for optimizing TEVG functionality when used in combination with other optimization strategies. Our results suggested that immobilized Jagged has variable consequences for TEVG properties, with some properties improving by becoming more native like, most notably compliance, while others become less native-like, such as collagen content and luminal radius. Moreover, immobilized Jagged was predicted not to substantially reduce the variation that resulted from patient-specific conditions for any TEVG property. Combined, these results suggest that small improvements in TEVG properties may be obtained by introducing immobilized Jagged, but these might come at the cost of making other properties worse and increasing variability. This has parallels with the challenge of finding a single scaffold parameter set to optimize all TEVG properties simultaneously [12]. Therefore, a combination of different optimization strategies may be needed to obtain optimal TEVG functionality. In this context, Notch may play a role in improving selected properties, such as increasing early compliance to reduce a compliance mismatch with the native artery [22, 63]. Overall, immobilized Jagged may be used to optimize TEVGs by improving selected properties but negative effects should be compensated for by changing other parameters. Our model may aid future optimization of in situ vascular tissue engineering by identifying the beneficial and detrimental effects of immobilized Jagged on TEVG properties.

In addition to patient-specific conditions affecting Notch signaling via mechanical stimuli, they may also have direct effects on Notch signaling. For example, diabetes impacts the expression of Notch receptors and ligands [72] and inflammatory stimuli can activate Notch [73]. Meanwhile, Notch signaling dynamics and immobilized Jagged ligands may also affect patient conditions, as it is known to play a role in macrophage regulation [74, 75], which may influence Notch-mediated tissue production (Eqs. (7) and (8)). This suggests that immobilized Jagged may interfere with the inflammatory response by interacting with macrophages and may therefore influence the efficacy of immobilized Jagged. So, while our model is able to predict the effects of patient-specific conditions on Notch via mechanical stimuli, future extensions should aim to incorporate additional avenues of crosstalk between Notch and patient characteristics.

Contrary to previous models [12, 27, 43, 44], but consistent with our previous study [35], we did not explicitly model temporal changes in true mass density of the mixture that may arise from degradation of (dense) polymeric scaffold in favor of (less dense) soft native tissue. Nevertheless, this limitation is unlikely to impact our study conclusions, drawn based on comparisons between different simulations. In particular, the decrease in true mass density would be present in all simulations, such that the trends observed in their differences would remain qualitatively equal.

In conclusion, to advance the field toward optimization of TEVGs and to investigate potential causes of TEVG variability, we developed a computational model to simulate the roles of Notch signaling and Notch manipulations on the G&R of TEVGs. This model predicted that changes in inflammatory production, scaffold degradation, and scaffold pre-stretch can elicit variability in TEVG properties via mechano-mediated production, partly driven by Notch mechano-regulation. Immobilized Jagged ligands were predicted to have both positive and negative effects as they make some TEVG properties more native-like, mainly compliance, and others less native-like, such as collagen content and luminal radius. These ligands should therefore only be adopted to improve selected TEVG properties when accompanied by other strategies to compensate for negative effects. Moreover, patient-specific characteristics should be considered when seeking optimization via immobilized Jagged ligands, as the sensitivity of TEVG properties to this intervention depends on patient-specific conditions. This framework sets the basis for future computational approaches building upon an increased biological understanding of the role of Notch in TEVG evolution and may be extended to incorporate other signaling pathways in a similar way.

## Data Availability

All data and computational codes are available at 10.4121/f1ca41cd-df90-4aac-9712-8fe4b1c25f80

## References

[1] Schoen, F. J., E. S. Fioretta, A. Mallone, et al. Vascular Tissue Engineering: Pathological Considerations, Mechanisms, and Translational Implications. In: Tissue-Engineered Vascular Grafts, Cham: Springer International Publishing, 2020, pp. 1–41.

[2] Gaharwar, A. K., I. Singh, and A. Khademhosseini. Engineered biomaterials for in situ tissue regeneration. *Nat Rev Mater*. 5:686–705, 2020. 10.1038/s41578-020-0209-x.

[3] Lee, A. Y., N. Mahler, C. Best, et al. Regenerative implants for cardiovascular tissue engineering. *Transl Res*. 163:321–341, 2014. 10.1016/j.trsl.2014.01.014. 24589506 10.1016/j.trsl.2014.01.014

[4] Wissing, T. B., V. Bonito, C. V. C. Bouten, and A. I. P. M. Smits. Biomaterial-driven in situ cardiovascular tissue engineering—a multi-disciplinary perspective. *npj Regen Med*. 2:18, 2017. 10.1038/s41536-017-0023-2. 29302354 10.1038/s41536-017-0023-2 PMC5677971

[5] Yuan, H., C. Chen, Y. Liu, et al. Strategies in cell-free tissue-engineered vascular grafts. *J Biomed Mater Res Part A*. 108:426–445, 2020. 10.1002/jbm.a.36825. 10.1002/jbm.a.36825 31657523

[6] Roh, J. D., R. Sawh-Martinez, M. P. Brennan, et al. Tissue-engineered vascular grafts transform into mature blood vessels via an inflammation-mediated process of vascular remodeling. *Proc Natl Acad Sci*. 107:4669–4674, 2010. 10.1073/pnas.0911465107. 20207947 10.1073/pnas.0911465107 PMC2842056

[7] Dimitrievska, S., and L. E. Niklason. Historical perspective and future direction of blood vessel developments. *Cold Spring Harb Perspect Med*. 8:a025742, 2018. 10.1101/cshperspect.a025742. 28348177 10.1101/cshperspect.a025742 PMC5685928

[8] Karakaya, C., J. G. M. van Asten, T. Ristori, et al. Mechano-regulated cell–cell signaling in the context of cardiovascular tissue engineering. *Biomech Model Mechanobiol*. 21:5–54, 2022. 10.1007/s10237-021-01521-w. 34613528 10.1007/s10237-021-01521-w PMC8807458

[9] Kumar, V. A., L. P. Brewster, J. M. Caves, and E. L. Chaikof. Tissue Engineering of Blood Vessels: Functional Requirements, Progress, and Future Challenges. *Cardiovasc Eng Technol*. 2:137–148, 2011. 10.1007/s13239-011-0049-3. 23181145 10.1007/s13239-011-0049-3 PMC3505086

[10] Smits, A. I. P. M., and C. V. C. Bouten. Tissue engineering meets immunoengineering: Prospective on personalized in situ tissue engineering strategies. *Curr Opin Biomed Eng*. 6:17–26, 2018. 10.1016/j.cobme.2018.02.006.

[11] Song, H.-H.G., R. T. Rumma, C. K. Ozaki, et al. Vascular tissue engineering: progress, challenges, and clinical promise. *Cell Stem Cell*. 22:340–354, 2018. 10.1016/j.stem.2018.02.009. 29499152 10.1016/j.stem.2018.02.009 PMC5849079

[12] Szafron, J. M., A. B. Ramachandra, C. K. Breuer, et al. Optimization of tissue-engineered vascular graft design using computational modeling. *Tissue Eng Part C Methods*. 25:561–570, 2019. 10.1089/ten.tec.2019.0086. 31218941 10.1089/ten.tec.2019.0086 PMC6791486

[13] Wu, W., R. A. Allen, and Y. Wang. Fast-degrading elastomer enables rapid remodeling of a cell-free synthetic graft into a neoartery. *Nat Med*. 18:1148–1153, 2012. 10.1038/nm.2821. 22729285 10.1038/nm.2821 PMC3438366

[14] Hibino, N., E. McGillicuddy, G. Matsumura, et al. Late-term results of tissue-engineered vascular grafts in humans. *J Thorac Cardiovasc Surg*. 139:431–436, 2010. 10.1016/j.jtcvs.2009.09.057. 20106404 10.1016/j.jtcvs.2009.09.057

[15] Talacua, H., A. I. P. M. Smits, D. E. P. Muylaert, et al. In situ tissue engineering of functional small-diameter blood vessels by host circulating cells only. *Tissue Eng Part A*. 21:2583–2594, 2015. 10.1089/ten.tea.2015.0066. 26200255 10.1089/ten.TEA.2015.0066

[16] Wu, Z., H. Luo, E. Thorin, et al. Possible role of Efnb1 protein, a ligand of Eph receptor tyrosine kinases, in modulating blood pressure. *J Biol Chem*. 287:15557–15569, 2012. 10.1074/jbc.M112.340869. 22393061 10.1074/jbc.M112.340869 PMC3346120

[17] Best, C. A., J. M. Szafron, K. A. Rocco, et al. Differential outcomes of venous and arterial tissue engineered vascular grafts highlight the importance of coupling long-term implantation studies with computational modeling. *Acta Biomater*. 94:183–194, 2019. 10.1016/j.actbio.2019.05.063. 31200116 10.1016/j.actbio.2019.05.063 PMC6819998

[18] Tara, S., H. Kurobe, M. W. Maxfield, et al. Evaluation of remodeling process in small-diameter cell-free tissue-engineered arterial graft. *J Vasc Surg*. 62:734–743, 2015. 10.1016/j.jvs.2014.03.011. 24745941 10.1016/j.jvs.2014.03.011

[19] Yang, X., J. Wei, D. Lei, et al. Appropriate density of PCL nano-fiber sheath promoted muscular remodeling of PGS/PCL grafts in arterial circulation. *Biomaterials*. 88:34–47, 2016. 10.1016/j.biomaterials.2016.02.026. 26943048 10.1016/j.biomaterials.2016.02.026

[20] Bergmeister, H., N. Seyidova, C. Schreiber, et al. Biodegradable, thermoplastic polyurethane grafts for small diameter vascular replacements. *Acta Biomater*. 11:104–113, 2015. 10.1016/j.actbio.2014.09.003. 25218664 10.1016/j.actbio.2014.09.003

[21] Duijvelshoff, R., A. di Luca, E. E. van Haaften, et al. Inconsistency in graft outcome of bilayered bioresorbable supramolecular arterial scaffolds in rats. *Tissue Eng Part A*. 27:894–904, 2021. 10.1089/ten.tea.2020.0185. 32873211 10.1089/ten.TEA.2020.0185

[22] Khosravi, R., K. S. Miller, C. A. Best, et al. Biomechanical diversity despite mechanobiological stability in tissue engineered vascular grafts two years post-implantation. *Tissue Eng Part A*. 21:1529–1538, 2015. 10.1089/ten.tea.2014.0524. 25710791 10.1089/ten.tea.2014.0524 PMC4426307

[23] Sugiura, T., S. Tara, H. Nakayama, et al. Novel bioresorbable vascular graft with sponge-type scaffold as a small-diameter arterial graft. *Ann Thorac Surg*. 102:720–727, 2016. 10.1016/j.athoracsur.2016.01.110. 27154152 10.1016/j.athoracsur.2016.01.110 PMC5002920

[24] Sugiura, T., S. Tara, H. Nakayama, et al. Fast-degrading bioresorbable arterial vascular graft with high cellular infiltration inhibits calcification of the graft. *J Vasc Surg*. 66:243–250, 2017. 10.1016/j.jvs.2016.05.096. 27687327 10.1016/j.jvs.2016.05.096 PMC5366287

[25] van Haaften, E., C. Bouten, and N. Kurniawan. Vascular mechanobiology: towards control of in situ regeneration. *Cells*. 6:19, 2017. 10.3390/cells6030019. 28671618 10.3390/cells6030019 PMC5617965

[26] Wise, S. G., M. J. Byrom, A. Waterhouse, et al. A multilayered synthetic human elastin/polycaprolactone hybrid vascular graft with tailored mechanical properties. *Acta Biomater*. 7:295–303, 2011. 10.1016/j.actbio.2010.07.022. 20656079 10.1016/j.actbio.2010.07.022

[27] Miller, K. S., R. Khosravi, C. K. Breuer, and J. D. Humphrey. A hypothesis-driven parametric study of effects of polymeric scaffold properties on tissue engineered neovessel formation. *Acta Biomater*. 11:283–294, 2015. 10.1016/j.actbio.2014.09.046. 25288519 10.1016/j.actbio.2014.09.046 PMC4256111

[28] Drews, J. D., V. K. Pepper, C. A. Best, et al. Spontaneous reversal of stenosis in tissue-engineered vascular grafts. *Sci Transl Med*. 2020. 10.1126/scitranslmed.aax6919. 32238576 10.1126/scitranslmed.aax6919 PMC7478265

[29] de Kort, B. J., S. E. Koch, T. B. Wissing, et al. Immuno-regenerative biomaterials for in situ cardiovascular tissue engineering—Do patient characteristics warrant precision engineering? *Adv Drug Deliv Rev*. 178:113960, 2021. 10.1016/j.addr.2021.113960. 34481036 10.1016/j.addr.2021.113960

[30] Baeten JT, Lilly B (2017) Notch signaling in vascular smooth muscle cells. In: Advances in Pharmacology. pp 351–382 10.1016/bs.apha.2016.07.002 PMC5964982 28212801

[31] Gridley, T. Notch signaling in vascular development and physiology. *Development*. 134:2709–2718, 2007. 10.1242/dev.004184. 17611219 10.1242/dev.004184

[32] Karakaya, C., M. C. van Turnhout, V. L. Visser, et al. Notch signaling regulates strain-mediated phenotypic switching of vascular smooth muscle cells. *Front Cell Dev Biol*. 10:1–19, 2022. 10.3389/fcell.2022.910503. 10.3389/fcell.2022.910503 PMC9412035 36036000

[33] Loerakker, S., O. M. J. A. Stassen, F. M. ter Huurne, et al. Mechanosensitivity of Jagged-Notch signaling can induce a switch-type behavior in vascular homeostasis. *Proc Natl Acad Sci*. 115:E3682–E3691, 2018. 10.1073/pnas.1715277115. 29610298 10.1073/pnas.1715277115 PMC5910818

[34] Morrow, D., C. Sweeney, Y. A. Birney, et al. Cyclic strain inhibits notch receptor signaling in vascular smooth muscle cells in vitro. *Circ Res*. 96:567–575, 2005. 10.1161/01.RES.0000159182.98874.43. 15705961 10.1161/01.RES.0000159182.98874.43

[35] van Asten, J. G. M., M. Latorre, C. Karakaya, et al. A multiscale computational model of arterial growth and remodeling including Notch signaling. *Biomech Model Mechanobiol*. 22:1569–1588, 2023. 10.1007/s10237-023-01697-3. 37024602 10.1007/s10237-023-01697-3 PMC10511605

[36] van Asten, J. G. M., T. Ristori, D. R. Nolan, et al. Computational analysis of the role of mechanosensitive Notch signaling in arterial adaptation to hypertension. *J Mech Behav Biomed Mater*. 133:105325, 2022. 10.1016/j.jmbbm.2022.105325. 35839633 10.1016/j.jmbbm.2022.105325 PMC7613661

[37] Carlson, M. E., M. S. O’Connor, M. Hsu, and I. M. Conboy. Notch signaling pathway and tissue engineering. *Front Biosci*. 12:5143–5156, 2007. 10.2741/2554. 17569636 10.2741/2554

[38] Tiemeijer, L. A., S. Sanlidag, C. V. C. Bouten, and C. M. Sahlgren. Engineering tissue morphogenesis: taking it up a Notch. *Trends Biotechnol*. 40:945–957, 2022. 10.1016/j.tibtech.2022.01.007. 35181146 10.1016/j.tibtech.2022.01.007 PMC7613405

[39] Zohorsky, K., and K. Mequanint. Designing biomaterials to modulate notch signaling in tissue engineering and regenerative medicine. *Tissue Eng Part B Rev*. 27:383–410, 2021. 10.1089/ten.teb.2020.0182. 33040694 10.1089/ten.TEB.2020.0182

[40] Beckstead, B. L., D. M. Santosa, and C. M. Giachelli. Mimicking cell–cell interactions at the biomaterial–cell interface for control of stem cell differentiation. *J Biomed Mater Res Part A*. 79A:94–103, 2006. 10.1002/jbm.a.30760. 10.1002/jbm.a.30760 16758464

[41] Putti, M., S. M. J. de Jong, O. M. J. A. Stassen, et al. A supramolecular platform for the introduction of Fc-fusion bioactive proteins on biomaterial surfaces. *ACS Appl Polym Mater*. 1:2044–2054, 2019. 10.1021/acsapm.9b00334. 31423488 10.1021/acsapm.9b00334 PMC6691680

[42] Putti, M., O. M. J. A. Stassen, M. J. G. Schotman, et al. Influence of the assembly state on the functionality of a supramolecular jagged1-mimicking peptide additive. *ACS Omega*. 4:8178–8187, 2019. 10.1021/acsomega.9b00869. 31172036 10.1021/acsomega.9b00869 PMC6545632

[43] Miller, K. S., Y. U. Lee, Y. Naito, et al. Computational model of the in vivo development of a tissue engineered vein from an implanted polymeric construct. *J Biomech*. 47:2080–2087, 2014. 10.1016/j.jbiomech.2013.10.009. 24210474 10.1016/j.jbiomech.2013.10.009 PMC3994188

[44] Szafron, J. M., R. Khosravi, J. Reinhardt, et al. Immuno-driven and mechano-mediated neotissue formation in tissue engineered vascular grafts. *Ann Biomed Eng*. 46:1938–1950, 2018. 10.1007/s10439-018-2086-7. 29987541 10.1007/s10439-018-2086-7 PMC6279560

[45] Humphrey, J. D., and K. R. Rajagopal. A constrained mixture model for growth and remodeling of soft tissues. *Math Model Methods Appl Sci*. 12:407–430, 2002. 10.1142/S0218202502001714.

[46] Latorre, M., and J. D. Humphrey. A mechanobiologically equilibrated constrained mixture model for growth and remodeling of soft tissues. *ZAMM—J Appl Math Mech / Zeitschrift für Angew Math und Mech*. 98:2048–2071, 2018. 10.1002/zamm.201700302. 10.1002/zamm.201700302 PMC6319907 30618468

[47] Baek, S., A. Valentín, and J. D. Humphrey. Biochemomechanics of cerebral vasospasm and its resolution: II. Constitutive relations and model simulations. *Ann Biomed Eng*. 35:1498–1509, 2007. 10.1007/s10439-007-9322-x. 17487585 10.1007/s10439-007-9322-x

[48] Boareto, M., M. K. Jolly, M. Lu, et al. Jagged-delta asymmetry in Notch signaling can give rise to a Sender/Receiver hybrid phenotype. *Proc Natl Acad Sci*. 112:E402–E409, 2015. 10.1073/pnas.1416287112. 25605936 10.1073/pnas.1416287112 PMC4321269

[49] Gibson, L., and M. F. Ashby. Cellular Solids: Structure and Properties. Cambridge: Cambridge University Press, 1999.

[50] Humphrey, J. D. Mechanisms of arterial remodeling in hypertension. *Hypertension*. 52:195–200, 2008. 10.1161/HYPERTENSIONAHA.107.103440. 18541735 10.1161/HYPERTENSIONAHA.107.103440 PMC2753501

[51] Bersi, M. R., R. Khosravi, A. J. Wujciak, et al. Differential cell-matrix mechanoadaptations and inflammation drive regional propensities to aortic fibrosis, aneurysm or dissection in hypertension. *J R Soc Interface*. 14:20170327, 2017. 10.1098/rsif.2017.0327. 29118111 10.1098/rsif.2017.0327 PMC5721146

[52] Naito, Y., Y.-U. Lee, T. Yi, et al. Beyond burst pressure: initial evaluation of the natural history of the biaxial mechanical properties of tissue-engineered vascular grafts in the venous circulation using a murine model. *Tissue Eng Part A*. 20:346–355, 2014. 10.1089/ten.tea.2012.0613. 23957852 10.1089/ten.tea.2012.0613 PMC3875183

[53] Cohn, D., and A. Hotovely Salomon. Designing biodegradable multiblock PCL/PLA thermoplastic elastomers. *Biomaterials*. 26:2297–2305, 2005. 10.1016/j.biomaterials.2004.07.052. 15585232 10.1016/j.biomaterials.2004.07.052

[54] Middleton JC, Tipton AJ (2000) Synthetic biodegradable polymers as orthopedic devices. 21. 10.1016/s0142-9612(00)00101-0 11055281

[55] Roh, J. D., G. N. Nelson, M. P. Brennan, et al. Small-diameter biodegradable scaffolds for functional vascular tissue engineering in the mouse model. *Biomaterials*. 29:1454–1463, 2008. 10.1016/j.biomaterials.2007.11.041. 18164056 10.1016/j.biomaterials.2007.11.041 PMC2375856

[56] Humphrey, J. D., and K. R. Rajagopal. A constrained mixture model for arterial adaptations to a sustained step change in blood flow. *Biomech Model Mechanobiol*. 2:109–126, 2003. 10.1007/s10237-003-0033-4. 14586812 10.1007/s10237-003-0033-4

[57] Irons, L., M. Latorre, and J. D. Humphrey. From transcript to tissue: multiscale modeling from cell signaling to matrix remodeling. *Ann Biomed Eng*. 2021. 10.1007/s10439-020-02713-8. 33415527 10.1007/s10439-020-02713-8 PMC8260704

[58] Langille, B. L. Remodeling of developing and mature arteries: endothelium, smooth muscle, and matrix. *J Cardiovasc Pharmacol*. 21:S11–S17, 1993. 7681126 10.1097/00005344-199321001-00003

[59] Nissen, R., G. J. Cardinale, and S. Udenfriendt. *Increased turnover of arterial collagen in hypertensive rats.* 75:451–453, 1978. 10.1073/pnas.75.1.451 PMC411267 272662

[60] Valentín, A., L. Cardamone, S. Baek, and J. Humphrey. Complementary vasoactivity and matrix remodelling in arterial adaptations to altered flow and pressure. *J R Soc Interface*. 6:293–306, 2009. 10.1098/rsif.2008.0254. 18647735 10.1098/rsif.2008.0254 PMC2659584

[61] Latorre, M., M. R. Bersi, and J. D. Humphrey. Computational modeling predicts immuno-mechanical mechanisms of maladaptive aortic remodeling in hypertension. *Int J Eng Sci*. 141:35–46, 2019. 10.1016/j.ijengsci.2019.05.014. 32831391 10.1016/j.ijengsci.2019.05.014 PMC7437922

[62] Yao, Y., J. Wang, Y. Cui, et al. Effect of sustained heparin release from PCL/chitosan hybrid small-diameter vascular grafts on anti-thrombogenic property and endothelialization. *Acta Biomater*. 10:2739–2749, 2014. 10.1016/j.actbio.2014.02.042. 24602806 10.1016/j.actbio.2014.02.042

[63] Khosravi, R., C. A. Best, R. A. Allen, et al. Long-term functional efficacy of a novel electrospun poly(glycerol sebacate)-based arterial graft in mice. *Ann Biomed Eng*. 44:2402–2416, 2016. 10.1007/s10439-015-1545-7. 26795977 10.1007/s10439-015-1545-7 PMC4938761

[64] Fukunishi, T., C. A. Best, T. Sugiura, et al. Tissue-engineered small diameter arterial vascular grafts from cell-free nanofiber PCL/chitosan scaffolds in a sheep model. *PLoS One*. 11:e0158555, 2016. 10.1371/journal.pone.0158555. 27467821 10.1371/journal.pone.0158555 PMC4965077

[65] Huang, R., X. Gao, J. Wang, et al. Triple-layer vascular grafts fabricated by combined E-jet 3D printing and electrospinning. *Ann Biomed Eng*. 46:1254–1266, 2018. 10.1007/s10439-018-2065-z. 29845412 10.1007/s10439-018-2065-z

[66] Huang, A. H., J. L. Balestrini, B. V. Udelsman, et al. Biaxial stretch improves elastic fiber maturation, collagen arrangement, and mechanical properties in engineered arteries. *Tissue Eng Part C Methods*. 22:524–533, 2016. 10.1089/ten.tec.2015.0309. 27108525 10.1089/ten.tec.2015.0309 PMC4921901

[67] Casella, S., A. Bielli, A. Mauriello, and A. Orlandi. Molecular pathways regulating macrovascular pathology and vascular smooth muscle cells phenotype in type 2 diabetes. *Int J Mol Sci*. 16:24353–24368, 2015. 10.3390/ijms161024353. 26473856 10.3390/ijms161024353 PMC4632754

[68] Lorentzen, K. A., S. Chai, H. Chen, et al. Mechanisms involved in extracellular matrix remodeling and arterial stiffness induced by hyaluronan accumulation. *Atherosclerosis*. 244:195–203, 2016. 10.1016/j.atherosclerosis.2015.11.016. 26671518 10.1016/j.atherosclerosis.2015.11.016

[69] Su, S.-C., Y.-J. Hung, C.-L. Huang, et al. Cilostazol inhibits hyperglucose-induced vascular smooth muscle cell dysfunction by modulating the RAGE/ERK/NF-κB signaling pathways. *J Biomed Sci*. 26:68, 2019. 10.1186/s12929-019-0550-9. 31492153 10.1186/s12929-019-0550-9 PMC6731603

[70] Yang, J., G. R. Gourley, A. Gilbertsen, et al. High glucose levels promote switch to synthetic vascular smooth muscle cells via lactate/GPR81. *Cells*. 13:236, 2024. 10.3390/cells13030236. 38334628 10.3390/cells13030236 PMC10854508

[71] Wang, Z., W. Zheng, Y. Wu, et al. Differences in the performance of PCL-based vascular grafts as abdominal aorta substitutes in healthy and diabetic rats. *Biomater Sci*. 4:1485–1492, 2016. 10.1039/C6BM00178E. 27537499 10.1039/c6bm00178e

[72] LaFoya, B., J. A. Munroe, M. M. Mia, et al. Notch: A multi-functional integrating system of microenvironmental signals. *Dev Biol*. 418:227–241, 2016. 10.1016/j.ydbio.2016.08.023. 27565024 10.1016/j.ydbio.2016.08.023 PMC5144577

[73] Fazio, C., and L. Ricciardiello. Inflammation and Notch signaling: a crosstalk with opposite effects on tumorigenesis. *Cell Death Dis*. 7:e2515–e2515, 2016. 10.1038/cddis.2016.408. 27929540 10.1038/cddis.2016.408 PMC5260996

[74] Hamilton Outtz, H., J. K. Wu, X. Wang, and J. Kitajewski. Notch1 deficiency results in decreased inflammation during wound healing and regulates vascular endothelial growth factor receptor-1 and inflammatory cytokine expression in macrophages. *J Immunol*. 185:4363–4373, 2010. 10.4049/jimmunol.1000720. 20739676 10.4049/jimmunol.1000720 PMC3887523

[75] Keewan, E., and S. A. Naser. The Role of Notch signaling in macrophages during inflammation and infection: implication in rheumatoid arthritis? *Cells*. 2020. 10.3390/cells9010111. 31906482 10.3390/cells9010111 PMC7016800

